# The Oncogene AF1Q is Associated with WNT and STAT Signaling and Offers a Novel Independent Prognostic Marker in Patients with Resectable Esophageal Cancer

**DOI:** 10.3390/cells8111357

**Published:** 2019-10-30

**Authors:** Elisabeth S. Gruber, Georg Oberhuber, Peter Birner, Michaela Schlederer, Michael Kenn, Wolfgang Schreiner, Gerd Jomrich, Sebastian F. Schoppmann, Michael Gnant, William Tse, Lukas Kenner

**Affiliations:** 1 Division of General Surgery, Department of Surgery, Comprehensive Cancer Center, Medical University of Vienna, 1090 Vienna, Austria; elisabeth.s.gruber@meduniwien.ac.at (E.S.G.); gerd.jomrich@meduniwien.ac.at (G.J.); sebastian.schoppmann@meduniwien.ac.at (S.F.S.); michael.gnant@meduniwien.ac.at (M.G.); 2 Institute of Pathology, Department of Experimental and Translational Pathology, Medical University of Vienna, 1090 Vienna, Austria; georg.oberhuber@patho.at (G.O.); peter.birner@meduniwien.ac.at (P.B.); michaela.schlederer@meduniwien.ac.at (M.S.); 3 PIZ—Patho im Zentrum GmbH, 3100 St. Poelten, Lower Austria, Austria; 4 Section of Biosimulation and Bioinformatics, Center for Medical Statistics, Informatics and Intelligent Systems (CeMSIIS), Medical University of Vienna, 1090 Vienna, Austria; michael.kenn@meduniwien.ac.at (M.K.); wolfgang.schreiner@meduniwien.ac.at (W.S.); 5 James Graham Brown Cancer Center, University of Louisville School of Medicine, Louisville, KY 40202, USA; 6 Division of Blood and Bone Marrow Transplantation, Department of Medicine, University of Louisville School of Medicine, Louisville, KY 40202, USA; 7 Christian Doppler Laboratory for Applied Metabolomics (CDL-AM), Medical University of Vienna, 1090 Vienna, Austria; 8 Institute of Laboratory Animal Pathology, University of Veterinary Medicine Vienna, 1210 Vienna, Austria; 9 CBmed Core Lab 2, Medical University of Vienna, 1090 Vienna, Austria

**Keywords:** AF1Q, MLLT11, WNT, STAT, esophageal cancer, prognosis

## Abstract

AF1q impairs survival in hematologic and solid malignancies. AF1q expression is associated with tumor progression, migration and chemoresistance and acts as a transcriptional co-activator in WNT and STAT signaling. This study evaluates the role of AF1q in patients with resectable esophageal cancer (EC). A total of 278 patients operated on for EC were retrospectively included and the expression of AF1q, CD44 and pYSTAT3 was analyzed following immunostaining. Quantified data were processed to correlational and survival analysis. In EC tissue samples, an elevated expression of AF1q was associated with the expression of CD44 (*p* = 0.004) and pYSTAT3 (*p* = 0.0002). High AF1q expression in primary tumors showed high AF1q expression in the corresponding lymph nodes (*p* = 0.016). AF1q expression was higher after neoadjuvant therapy (*p* = 0.0002). Patients with AF1q-positive EC relapsed and died earlier compared to patients with AF1q-negative EC (disease-free survival (DFS), *p* = 0.0005; disease-specific survival (DSS), *p* = 0.003); in the multivariable Cox regression model, AF1q proved to be an independent prognostic marker (DFS, *p* = 0.01; DSS, *p* = 0.03). AF1q is associated with WNT and STAT signaling; it impairs and independently predicts DFS and DSS in patients with resectable EC. Testing AF1q could facilitate prognosis estimation and provide a possibility of identifying the patients responsive to the therapeutic blockade of its oncogenic downstream targets.

## 1. Introduction

*AF1Q* was originally identified as an *MLL* fusion partner in acute myeloid leukemia patients with a t(1; 11)(q21; q23) translocation (*MLLT11*); here, multiple chromosomal translocations of the AF1q locus have been described [1]. Enhanced AF1q expression is associated with poor clinical outcome in hematologic and several solid malignancies, such as breast, thyroid as well as testicular cancer and neuroblastoma [2,3,4,5,6,7,8,9]. Further on, AF1q plays a role in cell differentiation and maintenance during neuronal development [10], and is involved in stem cell differentiation. Here, it promotes T cell development, and at the same time impairs B cell differentiation; in addition, CD34-enriched stem cells show high AF1q levels, which are diminished during cell differentiation [11]. We delineated the precise oncogenic function of AF1q in breast cancer models, where AF1q functions as transcriptional co-factor and interacts with the T-cell factor/lymphoid enhancer factor (TCF/LEF) transcriptional complex in the wingless-type MMTV integration site family (WNT) [3] and signal transducer and activator of transcription 3 (STAT3) [12] signaling pathway. These two core cancer pathways play a pivotal role in tumors of the gastrointestinal tract and are involved in tumor initiation, progression, metastases, and chemoresistance [13,14]. In colorectal cancer, WNT signaling is a major driver of oncogenicity, and AF1q has been reported to drive proliferation, migration, and invasion in vitro and in vivo [5,15]. Recently, the suppression of breast cancer dissemination has been subscribed to GATA3-driven miR29b induction, which reportedly inhibits AF1q [16]. Controversly, some studies reported γ-irradiation or doxorubicin-induced pro-apoptotic effects through Blc-2-antagonist of cell death (BAD) in liver and ovarian cancer cells that were mediated by AF1q [7,8]. However, the exact mechanisms that AF1q involves to promote cancer are largely unidentified.

Esophageal cancer (EC) is one of the 10 most common types of cancer and is responsible for more than half a million deaths worldwide [17]. Surgery is the treatment of choice in locally limited resectable disease, and surgical techniques have been extended recently to meet the resection challenges. For locally advanced stage cancers, preoperative treatment is recommended [18]. At the time of diagnosis, half of the patients are metastasized and might benefit from targeted therapies; still, randomized data in EC are scarce [18]. Since EC’s genetic landscape is very distinct and driver mutations differ largely among subgroups, the definition of therapeutic targets on the basis of a genetic analysis is challenging. However, several targets for blocking the proposed AF1q downstream pathways WNT and STAT have demonstrated satisfying tumor response in cancer patients [12,13,19,20,21,22].

The aim of this study was to elucidate the role of AF1q in resectable EC, which to date remains unclear. By correlating tumoral AF1q expression with the expression of downstream targets CD44 and STAT3 as well as clinciopathological parameters, we wanted to evaluate the oncogenic potential of AF1q and its prognostic value for postoperative survival. Our data provide evidence that AF1q is associated with both WNT and STAT signaling and serves as a prognostic factor in EC patients.

## 2. Materials and Methods

### 2.1. Study Population 

Patients diagnosed with cancer of the esophagus and the esophagogastric junction who had undergone surgery between 1992 and 2002 at the Division of General Surgery, Medical University of Vienna were included into the study under ethical approval from the local review board (‘Ethikkommission’) of the Medical University of Vienna, #1197/2019). In case of locally advanced stage cancer, patients received neoadjuvant treatment according to the latest clinical practice guidelines at the time of diagnosis followed by surgery. Histopathological staging was conducted according to the AJCC/UICC staging system [23]. Surgical specimens were processed to tissue microarrays (TMAs), in which each tumor was represented by triplicate core biopsies. The location of the tumors was assessed according to the rules published in the fourth edition of the World Health Organization (WHO) classification of gastrointestinal tumors [24]. In brief, all squamous carcinomas from the tubular esophagus and from the area of the esophagogastric junction were considered to be carcinomas of the esophagus. The proximal extent of the gastric folds was used as the landmark for the esophagogastric junction. Due to similar initiating factors and prognosis of adenocarcinomas of the distal esophagus and adenocarcinomas of the esophagogastric junction (AEG) as well as challenges in clear clinical and histopathological distinction, these two groups were merged before analysis [18]. Histological response to neoadjuvant chemotherapy was graded according to Mandard [25]. 

### 2.2. Immunohistochemistry

The expression of AF1q as well as CD44 (as bona fide WNT target gene) as a possible AF1q downstream target was evaluated in resected human EC specimens; pySTAT3 expression was available from previous studies [26]. Immunostainings were performed using a standard protocol. Paraffin sections were de-waxed, and for the antigen retrieval, a citrate buffer pH 6 (CD44) or a Tris/Ethylenediaminetetraacetic acid (EDTA) buffer pH 9 (AF1q) was used. After endogenous peroxidase blocking, avidin and biotin blocking steps were performed. The antibodies were incubated overnight at 4 °C in PBS+1% bovine serum albumin (BSA). The following antibodies were used: CD44 (Santa Cruz, sc-9960) in a 1:200 dilution and AF1q (Abcam, ab109016) in a 1:200 dilution. Slides were washed with phosphate buffered saline (PBS) the following day and incubated with polyvalent-secondary antibody (IDetect Super Stain System HRP, ID laboratories) and horseradish peroxidase (HRP; IDetect Super Stain System HRP, ID laboratories). Signals were visualized with 3-amino-9-ethylcarbazole (ID laboratories). After counterstaining with hemalaun, the slides were mounted. The specimen were analyzed by a board-certified pathologist. A specimen was considered as positive when at least 50% of tumor cells showed moderate or strong cytoplasmic marker expression. Antibody specificity has been confirmed in previous studies [3,12,26,27,28,29].

### 2.3. Statistical Analysis

In terms of experimental characteristics, data were described by mean (± standard deviation). Statistical differences were analyzed by a Student’s t-test. A two-sided *p*-value less than 0.05 was considered statistically significant.

In terms of patient and tumor characteristics, numerical data were described by median (range) and categorical variables were described by frequencies. In order to compare AF1q expression with patient and tumor characteristics, the chi-square test was applied as appropriate. In order to describe the correlation between AF1q and ordinal or continuous variables, the Spearman rank correlation coefficient (r_s_) was calculated. The inverse Kaplan–Meier method was used to estimate the median follow-up time [30]. Survival estimates were calculated by the Kaplan–Meier method with the log-rank test for group comparisons. DSS was defined as time from esophageal surgery until death from EC. DFS was defined as time from esophageal surgery until EC recurrence. In order to identify independent predictive factors for survival, established prognostic factors such as neoadjuvant therapy and histological tumor subtype, as well as TNM staging, tumor grading, and resection margin [18,31], were entered into a multivariable Cox regression model in addition to AF1q. MATLAB Version 9.6/R2019a (MathWorks) was used for all statistical calculations. For all analyses, a two-sided *p*-value less than 0.05 was considered statistically significant.

## 3. Results

### 3.1. AF1q Expression Analysis in Human Esophageal Cancer Samples

**Figure 1:**
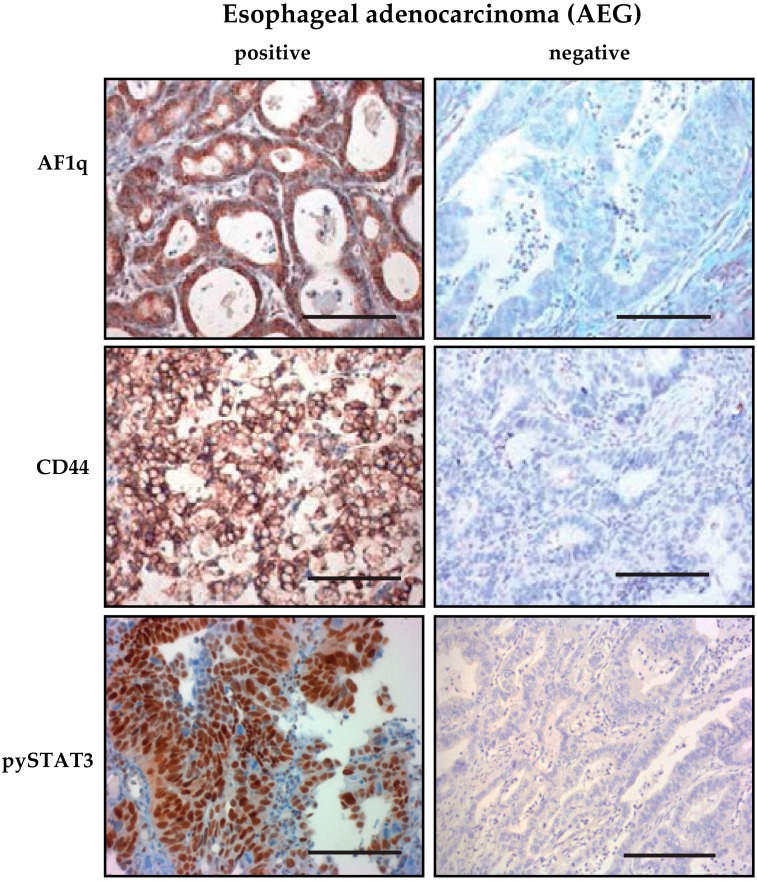
Examples of immunohistochemical (IHC) stained esophageal adenocarcinoma. Examples of AF1q expression in high-risk esophageal adenocarcinoma: Right example showing enhanced AF1q expression in high-risk adenocarcinoma vs. left example showing no AF1q expression in low-risk esophageal adenocarcinoma, size bar 100 µm.

In total, 278 patients with cancer of the esophagus and the esophagogastric junction as well as corresponding fully annotated tumor samples were retrospectively defined for analysis. The cohort consisted of 118 patients with esophageal squamous cell carcinoma (ESCC, 42.2%), 67 patients with esophageal adenocarcinoma (EAC, 24.1%) and 93 patients with adenocarcinoma of the esophagogastric junction (AEG, 33.5%); out of those, 138 tumor samples (49.6%) showed significant AF1q expression (ESCC, n = 54, 45.8%; EAC, n = 42, 62.7%; AEG, n = 42, 45.2%). An example of positive tumoral AF1q expression in EC is shown in Figure 1. Patient and tumor characteristics compared between AF1q-positive and AF1q-negative tumors are compiled in Table 1. In short, patients who received neoadjuvant therapy showed higher tumoral AF1q expression compared to patients who were resected upfront (*p* = 0.0002); histological response to neoadjuvant therapy did not correlate with AF1q expression (r_s_ = 0.22, *p* = 0.09). Patients with AF1q-positive tumors suffered from a higher rate of positive resection margins (R1) compared to patients with AF1q-negative tumors (*p* = 0.004). 

In order to evaluate an association of AF1q with WNT and STAT signaling, we correlated immunohistochemical (IHC) expression levels of CD44 (as bona fide WNT target gene) and tyrosine phosphorylated STAT3 (pYSTAT3) as possible AF1q downstream targets; we found a strong association of AF1q expression with both CD44 (n = 268, *p* = 0.004) as well as pYSTAT3 (n = 227, *p* = 0.0002) levels in EC (Table 2). Examples of positive tumoral CD44 as well as pYSTAT3 expression in EC are shown in Figure 1.

In order to explore AF1q-mediated dissemination features, we compared AF1q expression in lymph node metastases (n = 32) to AF1q expression in corresponding primary tumors and found a significant correlation (*p* = 0.016; Table 3).

### 3.2. Survival Analysis

**Figure 2:**
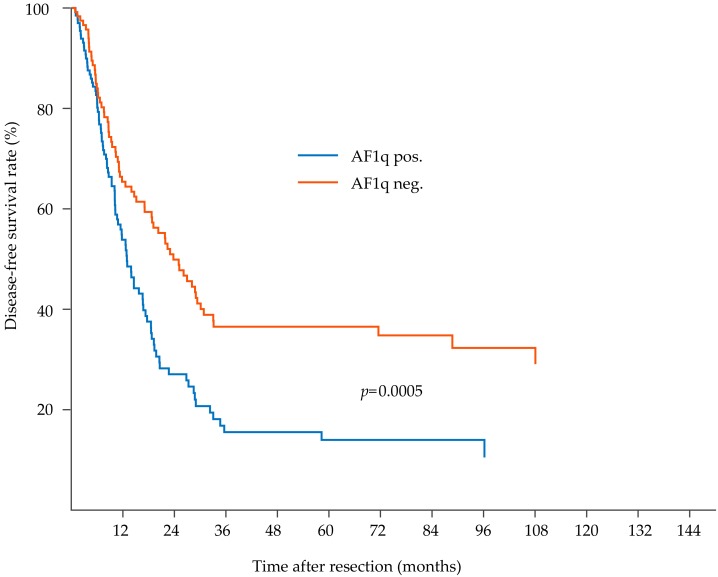
Kaplan–Meier analysis for disease-free survival in esophageal cancer (EC) patients. EC patients with high tumoral AF1q levels relapse earlier compared to patients with low or no tumoral AF1q expression (disease-free survival (DFS), log rank: *p* = 0.0005).

**Figure 3:**
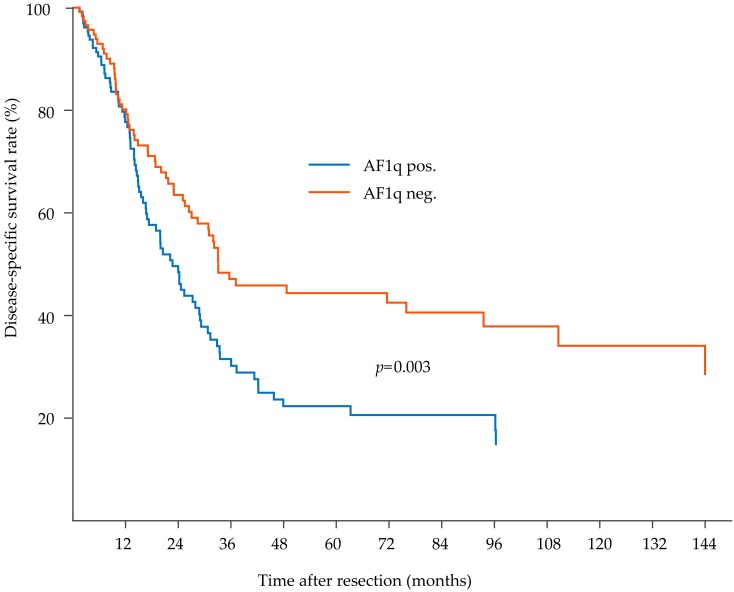
Kaplan–Meier analysis for disease-specific survival in esophageal cancer (EC) patients. EC patients with high tumoral AF1q die earlier compared to patients with low or no tumoral AF1q expression (disease-specific survival (DSS), log rank; *p* = 0.003).

During a median postoperative follow-up period of 71 months (range 52–90 months), 156 out of 278 (56.1%) patients relapsed, and 185 out of 278 (66.5%) patients had died of EC. The median DFS time was 17 months (range 13–20 months), and the median DSS time was 21 months (range 15–25 months). In patients with a high tumoral AF1q expression, the median DFS was 13 months (range 10–16 months) and the median DSS was 22 months (range 18–27 months) compared to 23 months (range 16–31 months) median DFS and 26 months (range 18–24 months) median DSS in patients with a low tumoral AF1q expression. Enhanced AF1q expression in the tumor proper resulted in significantly decreased DFS (Kaplan Meier/log rank; *p* = 0.0005; Figure 2) and DSS (Kaplan Meier/log rank, *p* = 0.003; Figure 3).

Cox regression analysis showed a 1.5 times higher risk for both disease recurrence and disease-specific death in patients with a high tumoral AF1q expression. Further prognostic factors were neoadjuvant therapy in regard to DFS (*p* = 0.0002), local tumor stage (DFS, *p* = 0.001 and DSS, *p* = 0.0004, respectively), regional lymph node metastases (DFS, *p <* 0.0001 and DSS, *p <* 0.0001, respectively), and resection margin (DFS, *p* = 0.02 and DSS, *p* = 0.01, respectively). In a multivariable Cox regression model, AF1q proved to be an independent factor for survival (DFS *p* = 0.01; DSS *p* = 0.03) next to the local tumor stage in regard to DSS (*p* = 0.007, respectively), regional lymph node metastases (DFS, *p <* 0.0001 and DSS, *p <* 0.0001, respectively), and histological tumor subtype (DFS *p* = 0.0004 and DSS, *p* = 0.003) as well as neoadjuvant therapy in regard to DFS (*p* = 0.005). The data of univariate and multivariable Cox regression analysis are compiled in Table 4.

## 4. Discussion

We show here for the first time the oncogenic potential as well as the utility of AF1q as a prognostic marker in esophageal cancer (EC). We demonstrate a significant association of AF1q expression with WNT as well as STAT3 signaling pathways, and show that tumoral AF1q expression impairs survival and serves as an independent prognostic factor in patients with resectable EC.

Well known for exerting multiple oncogenic functions, WNT and STAT3 have been shown to be of prognostic value in EC [13,14,26,32]. Both signaling pathways promote intestinal tumor growth and regeneration; interestingly, this can be reversed through gp130-JAK-STAT3 blockade [33]. These data imply that there might be a link between these two core cancer pathways. In fact, what WNT and STAT3 seem to share is AF1q as an activator of their target gene transcription. We previously reported that AF1q physically binds TCF/LEF and STAT3 and boosts their target gene transcription [3,12]. In breast cancer patients, the cooperation of AF1q with TCF7 led to the transcription of CD44 [3], which is a WNT target gene that is essentially involved in tumor progression and epithelial-to-mesenchymal transition [34,35]. In invasive breast cancer cells, AF1q further induced pYSTAT3 levels through the Src kinase-driven PDGFB/PDGFRB cascade [12]. Similarily to the findings in breast cancer, we here demonstrate an association of AF1q with both WNT and STAT signaling in the sense that the patients with AF1q-positive EC show enhanced tumoral levels of both proposed downstream targets CD44 and pYSTAT3.

AF1q expression is reportedly associated with metastatic spread in colorectal, lung, and breast cancer [5,12,36,37]. Since HER-2 is commonly expressed in EC, lung, and breast cancer [38,39,40,41], HER-2 might be another signaling pathway AF1q interacts with. However, further studies are needed to prove this concept.

We here demonstrate a correlation of AF1q expression in the local tumor with AF1q expression in the lymph node metastases of EC patients. In addition, AF1q-positive ECs show higher rates of positive resection margins compared to AF1q-negative ECs. We believe that the AF1q-induced co-activation of WNT and STAT signaling plays a key role in EC initiation, proliferation, and dissemination. Recently, Src kinase and JAK phosphorylation have been shown to facilitate STAT3 signaling [42], which further results in excessive proliferation and the malignant transformation of intestinal epithelial cells [43]. In invasive breast cancer cells, enhanced AF1q expression induced pYSTAT3 levels through the Src kinase-driven platelet-derived growth factor beta (PDGFB)/platelet-derived growth factor receptor beta (PDGFRB) cascade, which was reversible upon Src kinase blockade with protein phosphate 1 (PP1) or PDGFRB blockade using imatinib [12]. Consequently, patients with AF1q-positive EC are at exceptional risk to develop rapid disease progression that might respond to CD44 and STAT3 inhibition [13,19,20,21,22].

Conversely, AF1q has been reported to mediate the pro-apoptotic effects of cytotoxic agents such as doxorubicin, retinoic acid, or γ-irradiation through activation of the Blc-2-antagonist of cell death (BAD) [7,8,9]. In advanced stage EC, neoadjuvant (radio-)chemotherapy is applied to facilitate tumor shrinkage and consecutive R0 resection rates, and prevent recurrence [18,44]. Consistent with other findings, we showed an increased AF1q expression in patients treated with neoadjuvant therapy compared to treatment-naïve patients [7,8,12]. These findings support the fact that AF1q induction can be at least partly triggered by external factors such as cytotoxic agents and/or γ-irradiation [18]. Paradoxically, these data also indiciate that patients with AF1q-positive EC might suffer a disadvantage from currently applied oncologic treatment protocols due to fact that an enhanced induction of AF1q expression not only promotes EC dissemination, but might rather enforce EC’s metastic potential.

For a prognosis estimation of esophageal cancer, all staging efforts are recommended to be based on neoadjuvant therapy, histological tumor subtype, and TNM staging, as well as tumor grading and resection margin as important prognostic factors [18,31]. In this cohort of patients, AF1q proved to have a highly significant prognostic impact on both DFS and DSS in the univariate analysis. In fact, neoadjuvant therapy, histological tumor subtype, and regional lymph node status remained as independent prognostic factors for DFS and histological tumor subtype, local tumor stage, as well as regional lymph node status remained as independent prognostic factors for DSS in the model next to AF1q, respectively. Although none of these established prognostic factors correlated with tumoral AF1q expression, they demonstrate predominant importance for survival estimation in this selected cohort of patients with resectable esophageal cancer.

## 5. Conclusions

Our data underline the potency of AF1q to enforce and link the two major oncogenic pathways WNT and STAT3 involved in tumor initiation, progression, and dissemination. We demonstrate here for the first time a positive correlation between the expression of AF1q and the WNT and the STAT3 target genes CD44 and pYSTAT3 in EC, suggesting that AF1q may act as a cofactor for boosting the transcription of CD44 and pYSTAT3 in EC. Consequently, we demonstrate that patients with AF1q-positive EC relapse and die earlier. The association of AF1q with WNT and STAT3 signaling implicates various (combinatorial) targeting options such as CD44 and STAT3, JAK, and Src, as well as tyrosine kinases. Particularly, blockade of the AF1q/TCF7/CD44 regulatory axis as well as PDGF-B might hold therapeutic promise for patients with EC. Importantly, AF1q serves as an independent prognostic marker, and might add value to the estimation of resectability and disease progression. This study should prompt future clinical studies to validate these findings.

## Figures and Tables

**Table 1:** Patient and tumor characteristics compared between patients with AF1q-positive and AF1q-negative EC.

**Factor**	**Patients with EC, n = 278 (100.0)**	**Patients with AF1q-positive EC, n = 138 (49.6)**	**Patients with AF1q-negative EC, n = 140 (50.4)**	***p-*** **value**
Female sex	65 (23.4)	28 (43.1)	37 (56.9)	0.23 *
Male sex	213 (76.6)	110 (51.6)	103 (48.4)	
Age	63.3 (34–90)	63.8 (38–90)	63.9 (34–90)	rs = 0.03 ** (0.65)
Adiposity	16 (5.8)	10 (62.5)	6 (37.5)	0.11 *
Reflux	5 (1.8)	2 (40.0)	3 (60)	0.22 *
Neoadjuvant therapy	68 (24.5)	47 (69.1)	21 (30.9)	0.0002 *
**Factor**	**Patients with EC, n = 278 (100.0)**	**Patients with AF1q-positive EC, n = 138 (49.6)**	**Patients with AF1q-negative EC, n = 140 (50.4)**	***p-*** **value**
**Histological tumor subtype**				
**ESCC**	118 (42.4)	54 (45.8)	64 (54.2)	rs = 0.02 ** (0.88)
**AC**	160 (57.6)	84 (52.5)	76 (47.5)	
EAC	67 (24.1)	42 (62.7)	25 (37.3)	n.a.
AEG	93 (33.5)	42 (45.2)	51 (54.8)	
**AJCC/UICC tumor staging ESCC n = 118 (42.4)**				
IB	3 (2.5)	0 (0.0)	3 (100.0)	rs = 0.06 ** (0.55)
IIA	32 (27.1)	15 (46.9)	17 (53.1)	
IIIA	13 (11.0)	6 (46.2)	7 (53.8)	
IIIB	45 (38.1)	20 (44.4)	25 (55.6)	
IVA	19 (15.3)	10 (55.6)	8 (44.4)	
IVB	7 (5.9)	3 (42.9)	4 (57.1)	
**AJCC/UICC tumor staging EAC n = 67 (24.1)/AEG n = 93 (33.5)**				
IC	8 (5.0)	4 (50.0)	4 (50.0)	rs = 0.12 ** (0.14)
IIA	3 (1.9)	0 (0.0)	3 (100.0)	
IIB	21 (13.1.)	10 (47.6)	11 (32.4)	
IIIA	8 (5.0)	2 (25.0)	6 (75.0)	
IIIB	59 (36.9)	33 (55.9)	26 (44.1)	
IVA	61 (38.1)	35 (57.4)	26 (42.6)	
**Regional lymph nodes pN**				
N1	119 (44.1)	65 (54.6)	54 (45.4)	0.18 *
**Tumor grading G**				
G1	11 (4.0)	4 (36.4)	7 (63.6)	rs = 0.02 ** (0.69)
G2	132 (47.5)	66 (50.0)	66 (50.0)	
G3	135 (48.6)	68 (50.4)	67 (49.6)	
**Resection margin R**				
R0	228 (82.0)	104 (45.6)	124 (54.5)	0.004 *
R1	50 (18.0)	34 (68.0)	16 (32.0)	
**Histological response to neoadjuvant therapy**				
**none**	7 (10.3)	7 (10.3)	0 (0.0)	rs = 0.22 ** (0.09)
poor	30 (44.1)	21 (70.0)	9 (30.0)	
moderate	17 (25.0)	11 (64.7)	6 (35.3)	
good	4 (5.9)	2 (50.0)	2 (50.0)	

**Note.** Continuous variables are shown as median and range, categorical variables are expressed as absolute and relative numbers, n (%); adiposity is defined as BMI >30 kg/m2; EC—esophageal cancer; ESCC—esophageal squamous cell carcinoma; AC—adenocarcinomas; EAC—esophageal adenocarcinoma; AEG—adenocarcinomas of the esophagogastric junction; AJCC/UICC—American Joint Committee on Cancer/Union for International Cancer Control (https://cancerstaging.org/Pages/default.aspx; https://www.uicc.org); n.a.—not applicable; * chi-square test, ** Spearman correlation coefficient.

**Table 2:** Correlation of AF1q expression with proposed downstream target expression (CD44, pYSTAT3) in tumors of EC patients.

**Factor**	**Patients with AF1q-positive EC, n = 133 (49.6)**	**Patients with AF1q-negative EC, n = 135 (50.4)**	***p-*** **value**
**CD44, n = 268 (100)**			
positive, n = 94 (35.1)	58 (21.6)	36 (13.5)	0.004
negative, n = 174 (74.9)	75 (28.0)	99 (36.9)	
**Factor**	**Patients with AF1q-positive EC, n = 117 (51.5)**	**Patients with AF1q-negative EC, n = 110 (48.5)**	***p-*** **value**
**pYSTAT3, n = 227 (100)**			
positive, n = 101 (44.5)	66 (29.1)	35 (15.4)	0.0002
negative, n = 126 (55.5)	51 (22.4)	75 (33.1)	

**Note.** EC—esophageal cancer; variables are expressed as absolute and relative numbers, n (%).

**Table 3:** Correlation of enhanced AF1q expression in primary tumors and lymph node metastases of EC patients.

**Factor**	**AF1q-positive local EC, n = 18 (56.3)**	**AF1q-negative local EC, n = 14 (43.7)**	***p*-value**
**Lymph node metastases, n = 32 (100)**			
AF1q positive, n = 19 (59.4)	14 (43.8)	5 (15.6)	0.016
AF1q negative, n = 13 (40.6)	4 (12.5)	9 (28.1)	

**Note.** EC—esophageal cancer; variables are expressed as absolute and relative numbers, n (%).

**Table 4:** Univariable and multivariable Cox regression analysis in EC patients.

**Factor**	**Univariate *p*-Value**	**Multivariable *p*-Value**	**HR**	**95% CI Low**	**95% CI High**
**Disease-free survival**					
AF1q	0.0005	0.01	1.5	1.1	2.2
Neoadjuvant therapy	0.0002	0.005	1.7	1.2	2.6
Histological tumor subtype *	0.16	0.0004	1.9	1.3	2.7
Local tumor stage pT	0.001	0.07	n.a.		
Regional lymph nodes pN	<0.0001	<0.0001	2.3	1.6	3.2
Tumor grading G	0.13	0.30	n.a.		
Resection margin R	0.02	0.50	n.a.		
**Disease-specific survival**					
AF1q	0.003	0.03	1.5	1.0	2.1
Neoadjuvant therapy	0.10	0.83	n.a.		
Histological tumor subtype *	0.30	0.003	1.8	1.2	2.7
Local tumor stage pT	0.0004	0.007	1.9	1.2	3.0
Regional lymph nodes pN	<0.0001	<0.0001	2.2	1.5	3.2
Tumor grading G	0.22	0.48	n.a.		
Resection margin R	0.01	0.31	n.a.		

**Note.** EC—esophageal cancer; HR—hazard ratio; CI—confidence interval; HR and CR refer to multivariable model; * adenocarcinoma and squamous cell carcinoma.
