# Erratum: Gruber, E.S.; et al. The Oncogene AF1Q is Associated with WNT and STAT Signaling and Offers a Novel Independent Prognostic Marker in Patients with Resectable Esophageal Cancer. *Cells* 2019, *8*, 1357

**DOI:** 10.3390/cells9122724

**Published:** 2020-12-21

**Authors:** Elisabeth S. Gruber, Georg Oberhuber, Peter Birner, Michaela Schlederer, Michael Kenn, Wolfgang Schreiner, Gerd Jomrich, Sebastian F. Schoppmann, Michael Gnant, William Tse, Lukas Kenner

**Affiliations:** 1 Division of General Surgery, Department of Surgery, Medical University of Vienna, 1090 Vienna, Austria; elisabeth.s.gruber@meduniwien.ac.at (E.S.G.); gerd.jomrich@meduniwien.ac.at (G.J.); sebastian.schoppmann@meduniwien.ac.at (S.F.S.); 2 Comprehensive Cancer Center, Medical University of Vienna, 1090 Vienna, Austria; michael.gnant@meduniwien.ac.at; 3 Department of Experimental and Translational Pathology, Institute of Pathology, Medical University of Vienna, 1090 Vienna, Austria; georg.oberhuber@patho.at (G.O.); peter.birner@meduniwien.ac.at (P.B.); michaela.schlederer@meduniwien.ac.at (M.S.); 4 PIZ—Patho im Zentrum GmbH, 3100 St. Poelten, Austria; 5 Section of Biosimulation and Bioinformatics, Center for Medical Statistics, Informatics and Intelligent Systems (CeMSIIS), Medical University of Vienna, 1090 Vienna, Austria; michael.kenn@meduniwien.ac.at (M.K.); wolfgang.schreiner@meduniwien.ac.at (W.S.); 6 James Graham Brown Cancer Center, University of Louisville School of Medicine, Louisville, KY 40202, USA; 7 Division of Blood and Bone Marrow Transplantation, Department of Medicine, University of Louisville School of Medicine, Louisville, KY 40202, USA; 8 Christian Doppler Laboratory for Applied Metabolomics (CDL-AM), Medical University of Vienna, 1090 Vienna, Austria; 9 Institute of Laboratory Animal Pathology, University of Veterinary Medicine Vienna, 1210 Vienna, Austria; 10 CBmed Core Lab 2, Medical University of Vienna, 1090 Vienna, Austria

The authors wish to make the following change to their paper [1]: The Affiliations section is incorrect in the published paper with regards to the affiliation numbers of the authors. In addition, the e-mail address and phone number of the corresponding author has been updated.

The original version is:

Elisabeth S. Gruber ^1^, Georg Oberhuber ^2,3^, Peter Birner ^2^, Michaela Schlederer ^2^, Michael Kenn ^4^, Wolfgang Schreiner ^4^, Gerd Jomrich ^1^, Sebastian F. Schoppmann ^1^, Michael Gnant ^1^, William Tse ^5,6,^* and Lukas Kenner ^2,7,8,9,^*

^1^ Division of General Surgery, Department of Surgery, Comprehensive Cancer Center, Medical University of Vienna, 1090 Vienna, Austria; elisabeth.s.gruber@meduniwien.ac.at (E.S.G.); gerd.jomrich@meduniwien.ac.at (G.J.); sebastian.schoppmann@meduniwien.ac.at (S.F.S.); michael.gnant@meduniwien.ac.at (M.G.)

^2^ Department of Experimental and Translational Pathology, Institute of Pathology, Medical University of Vienna, 1090 Vienna, Austria; georg.oberhuber@patho.at (G.O.); peter.birner@meduniwien.ac.at (P.B.); michaela.schlederer@meduniwien.ac.at (M.S.)

^3^ PIZ—patho im zentrum GmbH, 3100 St. Poelten, Austria

^4^ Section of Biosimulation and Bioinformatics, Center for Medical Statistics, Informatics and Intelligent Systems (CeMSIIS), Medical University of Vienna, 1090 Vienna, Austria; michael.kenn@meduniwien.ac.at (M.K.); wolfgang.schreiner@meduniwien.ac.at (W.S.)

^5^ James Graham Brown Cancer Center, University of Louisville School of Medicine, Louisville, KY 40202, USA

^6^ Division of Blood and Bone Marrow Transplantation, Department of Medicine, University of Louisville School of Medicine, Louisville, KY 40202, USA

^7^ Christian Doppler Laboratory for Applied Metabolomics (CDL-AM), Medical University of Vienna, 1090 Vienna, Austria

^8^ Institute of Laboratory Animal Pathology, University of Veterinary Medicine Vienna, 1210 Vienna, Austria

^9^ CBmed Core Lab 2, Medical University of Vienna, 1090 Vienna, Austria

* Correspondence: william.tse@louisville.edu (W.T.); lukas.kenner@meduniwien.ac.at (L.K.); Tel.: +1-502-562-4363 (W.T.); +43-1-40400-51760 (L.K.)

This should be replaced with:

Elisabeth S. Gruber ^1,2^, Georg Oberhuber ^3,4^, Peter Birner ^3^, Michaela Schlederer ^3^, Michael Kenn ^5^, Wolfgang Schreiner ^5^, Gerd Jomrich ^1,2^, Sebastian F. Schoppmann ^1,2^, Michael Gnant ^2^, William Tse ^6,7,^* and Lukas Kenner ^3,8,9,10,^*

^1^ Division of General Surgery, Department of Surgery, Medical University of Vienna, 1090 Vienna, Austria; elisabeth.s.gruber@meduniwien.ac.at (E.S.G.); gerd.jomrich@meduniwien.ac.at (G.J.); sebastian.schoppmann@meduniwien.ac.at (S.F.S.)

^2^ Comprehensive Cancer Center, Medical University of Vienna, 1090 Vienna, Austria; michael.gnant@meduniwien.ac.at

^3^ Department of Experimental and Translational Pathology, Institute of Pathology, Medical University of Vienna, 1090 Vienna, Austria; georg.oberhuber@patho.at (G.O.); peter.birner@meduniwien.ac.at (P.B.); michaela.schlederer@meduniwien.ac.at (M.S.)

^4^ PIZ—Patho im Zentrum GmbH, 3100 St. Poelten, Austria

^5^ Section of Biosimulation and Bioinformatics, Center for Medical Statistics, Informatics and Intelligent Systems (CeMSIIS), Medical University of Vienna, 1090 Vienna, Austria; michael.kenn@meduniwien.ac.at (M.K.); wolfgang.schreiner@meduniwien.ac.at (W.S.)

^6^ James Graham Brown Cancer Center, University of Louisville School of Medicine, Louisville, KY 40202, USA

^7^ Division of Blood and Bone Marrow Transplantation, Department of Medicine, University of Louisville School of Medicine, Louisville, KY 40202, USA

^8^ Christian Doppler Laboratory for Applied Metabolomics (CDL-AM), Medical University of Vienna, 1090 Vienna, Austria; lukas.kenner@meduniwien.ac.at (L.K.)

^9^ Institute of Laboratory Animal Pathology, University of Veterinary Medicine Vienna, 1210 Vienna, Austria

^10^ CBmed Core Lab 2, Medical University of Vienna, 1090 Vienna, Austria

* Correspondence: wtse@metroHealth.org (W.T.); lukas.kenner@meduniwien.ac.at (L.K.); Tel.: +1-216-778-3845 (W.T.); +43-40400-51760 (L.K.)

We apologize for any inconvenience caused to the readers by this mistake. The manuscript will be updated and the original will remain online on the article webpage.
